# Role of Artificial Intelligence in Gastroenterology Training (2005–2025): Trends, Tools, and Challenges

**DOI:** 10.7759/cureus.90085

**Published:** 2025-08-14

**Authors:** Faiq Farooq, Muhammad Usman Tufail Warraich, Muhammad Moiz Ud Din, Nabeel Saleem, Muhammad Salman Asif, Haroon Khan

**Affiliations:** 1 Internal Medicine, Portsmouth Hospitals University NHS Trust, Portsmouth, GBR; 2 Gastroenterology and Hepatology, Portsmouth Hospitals University NHS Trust, Portsmouth, GBR; 3 Cardiology, Portsmouth Hospitals University NHS Trust, Portsmouth, GBR; 4 Orthopaedics and Trauma, Portsmouth Hospitals University NHS Trust, Portsmouth, GBR

**Keywords:** artificial intelligence in medicine, augmented reality (ar), endoscopic ultrasound (eus), gastroenterology training, simulation-based learning (sbl), simulation in medical education, virtual reality (vr)

## Abstract

Over the past two decades, gastroenterology training (GI training) has undergone a significant transformation through the integration of advanced technologies, particularly artificial intelligence (AI). The emergence of AI as a transformative tool has facilitated notable changes in how GI trainees acquire knowledge and skills. Key applications of AI in this context include simulation-based learning, diagnostic decision support, and procedural skill acquisition. These AI-driven innovations are increasingly recognised for enhancing learning efficiency, improving diagnostic accuracy, and building procedural confidence among trainees.

To explore the extent of AI’s impact on GI education, a comprehensive literature review was conducted. The search followed PRISMA guidelines and focused on peer-reviewed articles published between 2005 and 2025. Databases such as PubMed/NCBI, ScienceDirect, and the Cochrane Library were used, along with targeted searches in leading GI journals. The initial search yielded 312 records. After applying the inclusion and exclusion criteria, 22 studies were selected for final synthesis. These included randomised controlled trials, observational studies, systematic reviews, and narrative analyses.

The reviewed studies consistently demonstrated that AI-enhanced simulation tools, particularly those incorporating virtual reality (VR) and augmented reality (AR), played a pivotal role in procedural training. These tools offered immersive, risk-free environments that allowed trainees to practice and refine their technical skills before applying them in real-world clinical scenarios. AI also proved valuable in diagnostic decision support. Systems such as computer-aided detection (CADe) were shown to significantly increase lesion detection rates during endoscopic procedures, contributing to improved clinical decision-making and better patient outcomes. Additionally, AI-assisted technologies enhanced procedural training by supporting more precise biopsy targeting and facilitating lesion identification during endoscopic ultrasound (EUS).

As per our review, the evidence suggests that AI technologies are making meaningful contributions to GI training by improving diagnostic capabilities, streamlining the learning process, and supporting technical skill acquisition. However, despite these promising developments, further research is necessary. Future studies should include multi-centre randomised controlled trials and longitudinal evaluations to establish long-term efficacy. Furthermore, efforts toward global standardisation of AI training tools and equitable access are essential to ensure that these technologies benefit trainees across diverse clinical settings.

## Introduction and background

Currently, advanced technologies play an increasingly important role in gastroenterology training (GI training). It is the appropriate speciality when considering the role of artificial intelligence (AI) and simulation-based training due to its substantial reliance on visual pattern recognition (e.g., in endoscopy), procedural skill acquisition, and the potential for simulation to safely replicate complex, high-stakes scenarios for repetitive practice and feedback. Simulation-based learning refers to the use of virtual or physical models to recreate clinical procedures in a controlled, risk-free environment.

While recent reviews have effectively highlighted the clinical capabilities of AI in GI training [1], this study provides a uniquely comprehensive perspective focused on the educational implications over a 20-year period. This wider scope allows for vertical studies to be included and gives us a sense of how AI in GI training is evolving. By categorising AI applications across simulation, diagnostics, and procedural domains, and critically engaging with ethical and cognitive challenges, this review contributes a training-centric lens that has been underrepresented in prior literature. Additionally, emphasis has also been directed at global standardisation and adaptive learning frameworks, which address practical and policy-level gaps, offering strategic direction for future curricular integration of AI in gastroenterology education (GI education). 

Early on, simulation-based learning, particularly virtual reality (VR) endoscopy simulators, was adopted to supplement the traditional apprenticeship model, allowing trainees to practice endoscopic skills in a risk-free environment [1-3]. This laid the groundwork for integrating AI tools into training. In recent years, AI has begun transforming GI training; for instance, endoscopic simulation is now aided by real-time feedback through VR and augmented reality (AR) interfaces by assisting with tasks such as polyp detection and lesion characterisation using computer-aided detection (CADe) systems, which are AI algorithms designed to aid identification of abnormalities [4,5]. These capabilities directly impact how gastroenterology fellows learn procedural and diagnostic skills and have been shown to improve the pace of learning in novice trainees [4]. At the same time, the introduction of AI raises considerations such as cognitive overload for learners, over-reliance on AI support, magnification of AI bias, and disparities in access to such technology across training programs and the need for global standardisation [6-9]. 

AI applications in GI training are mainly observed in three domains: simulation-based learning, diagnostic decision support, and procedural training [2,4,8]. This literature review explores the breadth of AI integration in GI training between 2005 and 2025 in these areas, along with key limitations and future directions. 

Methods 

A systematic literature review was conducted in accordance with PRISMA 2020 guidelines [10]. The search targeted peer-reviewed journal articles published between January 2005 and May 2025 that addressed the use of AI in GI education or training. The following databases were searched: PubMed, ScienceDirect, and The Cochrane Library. Additionally, journal websites for *Gastroenterology* (published by the American College of Gastroenterology), *Gut* (published by the BMJ Publishing Group), *Clinical Gastroenterology and Hepatology* (Elsevier), *Journal of Gastroenterology* (Springer), and the *American Journal of Gastroenterology* (Wolters Kluwer) were manually searched for relevant articles. Manual searches on journal sites were conducted using combinations of the terms: "artificial intelligence," "machine learning," "gastroenterology education," "endoscopy training," and "simulation in GI fellowship." All retrieved records were imported into a reference manager, and duplicates were removed prior to screening.

Inclusion Criteria

Peer-reviewed studies, reviews, or consensus guidelines focusing on AI applications in the education or skills training of gastroenterology specialists (e.g., fellows or residents) were included in this study. Studies addressing any aspect of GI training were also included, including clinical decision-making, diagnostic interpretation, endoscopic procedure training, and simulation-based education, where an AI-based tool or method was evaluated or discussed. Publications from 2005 through 2025 in English were included.

Exclusion Criteria

Articles that concluded that the use of AI in clinical care would have no impact on GI trainee education were excluded. Additionally, non-peer-reviewed content (commentaries, letters, conference abstracts) was also excluded unless they provided unique insights from reputable sources. No exclusions were made based on study design; both experimental studies (e.g., randomised trials) and descriptive or review articles were included if they met the above inclusion criteria. 

**Figure 1:**
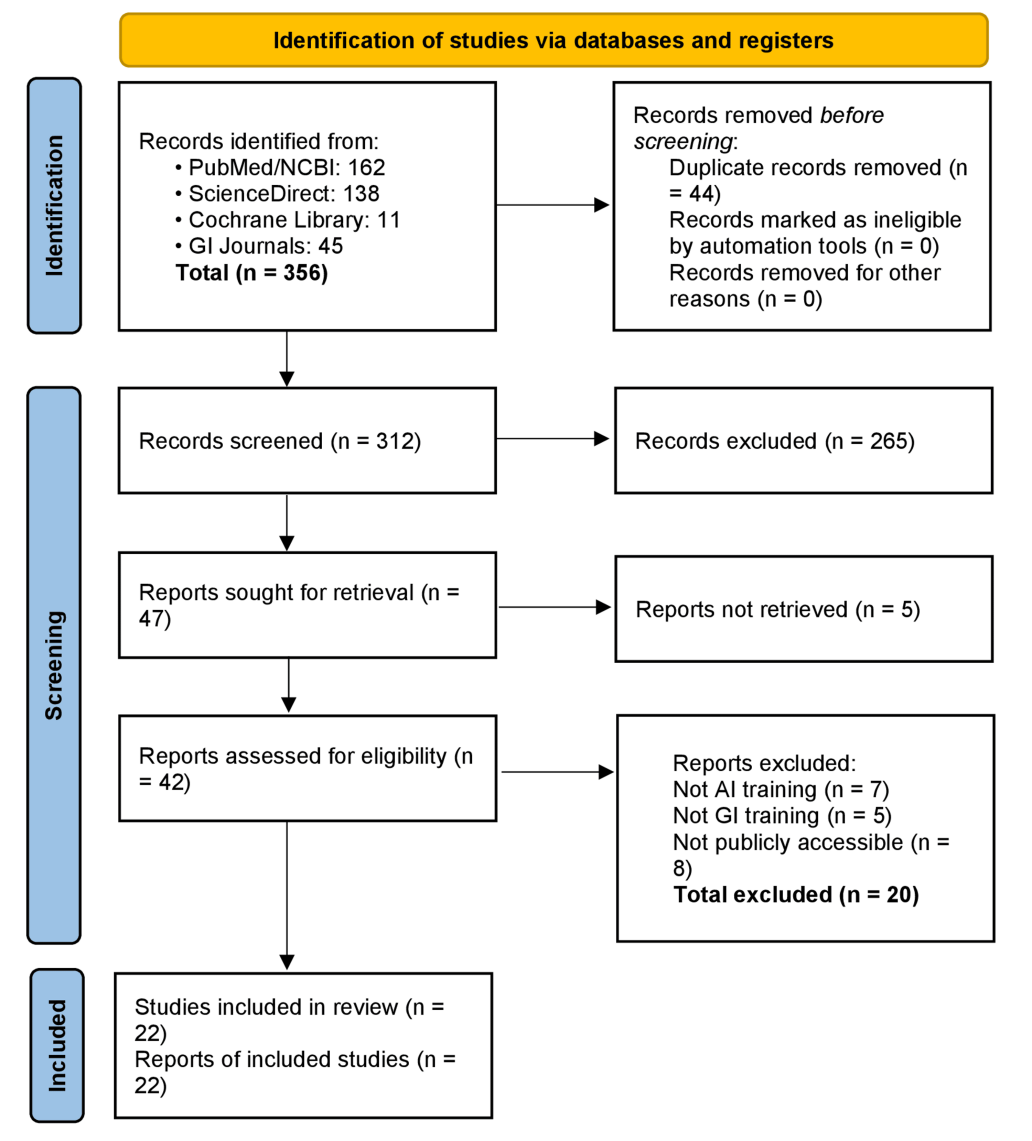
PRISMA flow diagram of literature search and selection

Using this strategy, a total of 312 records (after removing duplicates) were identified across the databases. Titles and abstracts were screened for relevance, yielding 47 articles for full-text evaluation. After applying the inclusion criteria, 22 studies were finally included in the review. Figure 1 illustrates the study selection process in a PRISMA flow diagram. Key data from the included studies, such as AI application domain, study design, and main findings-were extracted and are summarised in the following section. Due to the heterogeneity of study designs and outcomes, a narrative synthesis was conducted instead of a meta-analysis. Some studies included in the review do not directly pertain to GI training but were deemed relevant for providing contextual evidence on broader themes such as simulation-based training and skill acquisition. 

## Review

Overview of included studies 

Of the 22 included publications, there were four randomised controlled trials (RCTs), three systematic reviews/meta-analyses, two observational studies, and 13 narrative reviews or consensus papers. The majority of studies (≈77%) were published in the last five years (2021-2025), reflecting a growing interest in AI applications in GI training. The included literature covered three major thematic areas: simulation-based training tools, AI-driven diagnostic support for trainees, and AI in procedural skill acquisition. Table 1 provides a summary of studies on AI applications in GI training (2005-2025). Of note, the article also includes studies that are not directly related to the use of AI in GI training but are important in providing contextual relevance, such as the success of simulation-based training in aviation [11]. These have been excluded from Table 1.

**Table 1:** Summary of Key Studies on AI Applications in GI Training (2005–2025) Abbreviations: AI: artificial intelligence; VR: virtual reality; AR: augmented reality; CADe: computer-aided detection; EGD: esophagogastroduodenoscopy; EUS: endoscopic ultrasound; ERCP: endoscopic retrograde cholangiopancreatography; GI: gastrointestinal

Year	Study	Study Design	AI Application in Training	Key Findings
2025	Kang et al. [1]	Narrative review	General AI applications	Positive impact on trainee education, decision-making, and skill enhancement.
2019	Khan et al. [2]	Systematic review and meta-analysis	VR simulation	Improved endoscopic performance and training outcomes.
2006	Cohen et al. [3]	Randomized controlled trial	VR simulator for colonoscopy	Accelerated competency acquisition in trainees.
2021	Huang et al. [4]	Randomized controlled trial	Computer-assisted EGD	Reduced learning curve and improved procedural quality.
2024	Lau et al. [5]	Randomized controlled trial	Real-time polyp detection (CADe)	Enhanced adenoma detection among trainees.
2024	Campion et al. [6]	Narrative review	Human-AI interaction in GI endoscopy	Identified cognitive biases and interaction challenges.
2025	Ramoni et al. [7]	Narrative review	Ethical and diagnostic AI challenges	Identified ethical and diagnostic integration issues.
2024	Ahmed et al. [8]	Perspective article	Generative AI tools	Identified potential educational benefits and risks.
2024	Yuan et al. [9]	Retrospective observational study	EGD anatomical site classification	High accuracy in anatomical classification.
2018	Bhushan et al. [12]	Review article	AR and VR endoscopic training	Enhanced procedural training outcomes.
2018	Vilmann et al. [13]	Randomized controlled trial	Computerized colonoscopy feedback	Improved colonoscopy trainee performance.
2014	Blackburn and Griffin [14]	Review article	Simulation training	Reduced errors through simulation-based learning.
2023	Tsai et al. [15]	Retrospective observational study	Barrett’s oesophagus detection	Improved detection accuracy with AI.
2025	Dhali et al. [16]	Systematic review & meta-analysis	AI-assisted capsule endoscopy	Improved detection rates of small bowel lesions.
2022	Quan et al. [17]	Multi center pilot study	Real-time AI polyp detection	Proven effectiveness in clinical real-time application.
2022	Spadaccini et al. [18]	Narrative review	Enhanced EUS imaging	Improved imaging capabilities for pancreatic lesions.
2022	Dahiya et al. [19]	Narrative review	EUS in pancreatic cancer	Enhanced diagnostic precision and training insights.
2025	Araújo et al. [20]	Narrative review	AI in EUS and ERCP	Potential significant enhancement in diagnostic abilities.
2024	Gong et al. [21]	Systematic review	Large language models	Effectively supported clinical reasoning and educational documentation.
2022	Kader et al. [22]	Survey study	Perceptions of AI by clinicians	Identified perceptions, benefits, and barriers to AI adoption.
2023	Dhaliwal and Walsh [23]	Narrative review	AI in pediatric endoscopy	Reviewed current and future AI applications.
2023	Ahmad et al. [24]	Review article	AI in inflammatory bowel disease	Improved clinical practice and educational approaches.

Discussion 

The interest in AI has surged in the past few years. However, in medicine, technologies that incorporate AI have been around for much longer. It's important to examine how AI has shaped the GI training curriculum over the past two decades to extrapolate and ideally steer the future of AI-based GI training transformation. Most work that has been done to demonstrate the use of AI in GI training can be categorised into simulation-driven education, decision-making assistance, and the development of hands-on procedural competencies. Below, we discuss the benefits and limitations of AI in GI training in these domains.

Simulation-Based Learning 

Two commonly mentioned types of simulation are VR and AR. VR is a completely digitally constructed field of view; however, AR is a digital overlay on top of a real-world video feed. Simulation technology, including AR and VR, is used in training in a wide variety of high-stakes professions, such as aviation, where it allows learners to gain hands-on experience with equipment and procedures in a consequence-free environment. This approach enables trainees to improve performance and decision-making skills without the risks associated with real-world errors. For example, an RCT by Taylor et al. demonstrated that simulation-based training using personal computer aviation training devices (PCATDs) significantly improved instrument flying skills among private pilots, supporting the transfer of simulator-acquired skills to real flight and highlighting the value of simulation in enhancing training outcomes and flight safety [11]. 

Much like aviation, GI training also demands rigorous, off-the-field and high-fidelity simulation-based learning to ensure technical competence and patient safety in real-world procedures. Procedures like endoscopy can be high-stakes, and the risk of complications is related to the operator’s level of expertise. In that, VR-based simulators have shown promise in enhancing endoscopic skills among trainees by providing immersive, risk-free practice environments. For instance, Khan et al. conducted a high-quality meta-analysis examining the effectiveness of VR simulation for gastrointestinal endoscopy training [2]. Their review found that those who trained with VR simulators were more likely to successfully complete endoscopic procedures independently compared to those who received no simulation training at all. The analysis also showed that VR-trained participants demonstrated better mucosal visualisation and higher overall performance ratings. However, when VR simulation was compared directly to traditional patient-based training, the results were more nuanced: there was no clear superiority of VR in terms of overall competency or independent procedure completion. Importantly, the review emphasised that VR simulation is most beneficial when used as a structured part of a comprehensive curriculum, rather than as a standalone or unstructured tool. This suggests that while VR simulation is a powerful supplement for GI trainees - helping them build foundational skills and confidence - it should be integrated thoughtfully alongside real-life patient experience for optimal educational outcomes. 

Similarly, Bhushan et al. explored emerging applications of AR in endoscopic education [12]. They described how AR technology can project helpful digital overlays, such as anatomical references or procedural tips, onto the live endoscopic image, which assists learners in navigating complex anatomy and making informed decisions during procedures. This added layer of guidance helps trainees develop a stronger sense of spatial orientation and more quickly hone the technical aspects of endoscopy. The addition of real-time AI-driven feedback during endoscopic procedures has also been shown to accelerate skill acquisition; Vilmann et al. found that computerised feedback during colonoscopy training reduced error rates and increased learning efficiency among trainees [13]. Additionally, trainees who received computerised feedback spent more time practising and demonstrated more effective training patterns, such as reaching the caecum more frequently during practice sessions. The study concluded that automated, objective feedback not only motivates trainees but also enhances learning efficiency and skill acquisition in GI endoscopy simulation. 

Another aspect to consider is the realism or fidelity of simulation and its impact on outcomes. Blackburn and Griffith demonstrated that increasing fidelity alone leads to superior outcomes in real patient care [14]. With time, as various companies are investing heavily in improving simulation experience, including technology giants such as Meta and Apple, fidelity is expected to improve. Once simulation technology improves, it's only a matter of time before it's picked up by companies designing simulation training tools in medicine.

Diagnostic Decision Support 

AI-based diagnostic tools have improved detection rates and decision-making accuracy in endoscopy. Lau et al. conducted a prospective, randomised study evaluating the impact of computer-aided detection (CADe) systems on the performance of GI trainees during colonoscopy [5]. The study found that trainees using AI-based CADe technology had significantly higher adenoma detection rates compared to those performing standard colonoscopy without AI assistance. The CADe system provided real-time visual alerts when potential polyps were present, allowing trainees to identify and assess lesions they might otherwise have missed. This not only improved diagnostic accuracy but also contributed to a more thorough examination, which is critical for early cancer detection and prevention. 

Furthermore, Tsai et al. developed and validated an AI system trained to improve the detection of Barrett's oesophagus, a known precursor to oesophageal adenocarcinoma [15]. In their study, the AI model was trained using annotated images from experienced endoscopists and then tested on a separate set of cases with histological confirmation. The AI system achieved high diagnostic performance, with an accuracy, specificity, and sensitivity of over 90% for identifying Barrett's oesophagus. Similarly, with other conditions such as Crohn’s disease, Dhali et al. observed that AI in capsule endoscopy enhanced lesion detection rates with better sensitivity, and positive predictive values [16]. Quan et al. also found that AI-based polyp localisation increased the detection of flat lesions, which are traditionally more challenging to identify [17].

AI has also been noted to have a role in endoscopic ultrasound (EUS) training. EUS is a technique that combines endoscopy and ultrasound to visualise structures in the gastrointestinal tract (GI tract). Spadaccini et al. highlighted that AI-assisted endoscopic ultrasound improved lesion identification accuracy [18]. This improvement is echoed in studies like Dahiya et al., where AI-driven EUS was shown to increase diagnostic accuracy in pancreatic cyst evaluation [19]. 

Building on these advancements, AI's integration into more complex endoscopic procedures - such as EUS and endoscopic retrograde cholangiopancreatography (ERCP) - is also demonstrating significant clinical value. Recent studies have shown that AI-driven systems can accurately differentiate between benign and malignant pancreaticobiliary lesions, assist in real-time anatomical recognition, and predict procedural difficulty, thereby streamlining workflow and improving diagnostic consistency. For example, models trained to identify ampullary landmarks or assess cannulation difficulty have matched expert performance, while deep learning algorithms analysing EUS images have surpassed traditional guidelines in predicting malignancy risk in pancreatic cysts. These developments underscore AI's growing role not only in enhancing trainee performance but also in supporting experienced clinicians through complex decision-making processes across a wider range of gastrointestinal endoscopy modalities [20]. 

These studies demonstrate the potential for transforming GI training and education by providing objective decision support. Such tools can not only train novice operators in recognising subtle mucosal changes and improve their diagnostic confidence and accuracy, but also have the potential to standardise training outcomes and reduce inter-operator variability. 

Procedural Skill Acquisition 

Yuan et al. developed and validated an AI model designed to automatically classify anatomical sites in oesophagogastroduodenoscopy (OGD) images [9]. Their system was trained on a large, annotated dataset and demonstrated high accuracy in distinguishing between various anatomical regions of the upper GI tract, such as the oesophagus, stomach, and duodenum. The AI model achieved an overall accuracy exceeding 95%, with strong sensitivity and specificity for each anatomical location. 

In the context of GI training and education, this technology offers substantial benefits. Accurate identification of anatomical landmarks is a fundamental skill for endoscopists, and errors in this area can lead to missed lesions or procedural complications. By providing real-time, automated feedback on anatomical site recognition, the AI system developed by Yuan et al. can help trainees build procedural expertise more efficiently and with greater confidence. This also has the potential to reduce the time of direct supervision by an expert endoscopist required for a new operator to achieve competence, which can be financially beneficial. Furthermore, such AI-driven tools can standardise the learning process, ensuring that all trainees, regardless of their prior experience, achieve a consistent level of competency in endoscopic navigation and anatomical identification. Additionally, in centres with limited affordability and availability of expert mentors, this medium of practical training could be a lucrative adjunct to conventional training. 

Ethical and Practical Considerations 

Like all transformative technologies, AI in gastroenterology brings with it a host of ethical considerations. Introducing these concerns early in the overall conversation about AI in medicine is not merely prudent - it’s essential. By proactively raising awareness and encouraging open dialogue, we ensure that ethical reflection becomes a core component of how these technologies evolve. In doing so, we shape a future where innovation is guided not just by what is possible, but by what is responsible, equitable, and aligned with the values of patient-centred care. Ramoni et al. recognise several ethical challenges arising from the integration of AI into GI training [7]. One major concern is the risk of de-skilling among trainees, as increasing reliance on AI-driven diagnostic and procedural tools may diminish the development of independent clinical judgment and technical expertise. This is particularly relevant in GI training, where nuanced decision-making and hands-on skills are critical for safe and effective patient care. Over-reliance on AI can shift the role of clinicians from active problem-solvers to passive overseers of machine-generated recommendations, potentially weakening their ability to manage complex or atypical cases without technological assistance or perhaps losing the confidence to operate without AI.

Data privacy and algorithmic bias are also significant ethical considerations [7]. AI models in gastroenterology are often trained on large datasets that may not be fully representative of diverse patient populations, leading to the risk that diagnostic accuracy and recommendations may be less reliable for underrepresented groups. This can exacerbate existing healthcare disparities if not addressed through rigorous dataset curation and continuous validation. Furthermore, the use of sensitive patient data in AI development necessitates robust data protection measures and transparency to maintain trust and comply with regulations. 

In a hypothetical future where there's indeed over-reliance on AI and de-skilling of human operators, there may be a lack of accuracy in clinical acumen among human operators, leading to exaggerated manifestation of known shortcomings of AI systems, such as algorithmic and training bias. Additionally, if the final clinical judgement is from AI itself, who is accountable for that decision-AI or the human operator? If it is indeed AI, what is the penalty for a wrong decision? Would we "suspend its licence to practice" for a duration of time and cripple the very system that relies on it? That, while AI has absolutely no sense of the emotional or financial bearing of this “consequence”, would it really be a penalty against the AI? While one might argue that responsibility for errors made by AI should rest with the developers or companies that train these systems, the current paradigm frames AI as a tool of clinical "assistance" rather than as an autonomous decision-maker. In theory, AI outputs are intended to inform rather than dictate decisions, with final clinical judgement firmly in the hands of the practitioner, with whom the accountability of decisions will likely continue to remain. As AI systems become increasingly embedded in diagnostic and procedural workflows, there is a growing risk that clinicians, especially those in training, will absorb the assumptions, limitations, and biases of these tools as clinical truths. This is not mere passive exposure but a potential recalibration of clinical reasoning. In such a landscape, accountability paradoxically remains with the clinician. A future where there is indeed over-reliance among clinicians on AI systems that frame their input as "supplementary", clinicians may be more prone to legal implications. The issue to be mindful of, then, is not that AI will overtly replace the clinician, but that it will quietly rewire clinical cognition and amplify bias or small errors to a much larger scale. Perhaps in the future, mistakes will only be followed by investigation and improvement of the AI algorithm, skipping consequences straight to improvement of the system. Therefore, making it a potentially temporary issue (if at all), as AI systems are capable of improving quite rapidly, perhaps the overall rate of procedural complications will fall because of improvement in GI training and procedural execution, therefore, overall reducing consequences for everyone involved, including patients, clinicians, and other staff and technological systems.

Finally, the introduction of AI into training environments requires clear ethical guidelines to ensure that these technologies augment rather than replace human expertise, and that trainees remain actively engaged in developing core competencies. Addressing these challenges is essential for the responsible adoption of AI in GI education, ensuring that technological advancements translate into equitable, safe, and effective patient care. 

It's important to note that none of the aforementioned ethical pitfalls should discourage us from adopting, researching, integrating, or developing AI infrastructure in GI training and in healthcare more broadly. Instead, it should encourage us to be acutely aware of these considerations and have a structured training pathway that balances AI integration with traditional learning methods to avoid dependency and ensure robust clinical judgment development. Table 2 shows the various technologies used in GI education and training, their benefits, and drawbacks [2,3,5-9,12,13,15,17-22]. 

**Table 2:** Various technologies used in gastroenterology practice and training, their strengths and limitations. AI: artificial intelligence; VR: virtual reality; AR: augmented reality; CADe: computer-aided detection; EUS: endoscopic ultrasound; GI: gastrointestinal

AI Tool	Primary Use	Strengths	Limitations
VR simulation	Endoscopy training, hands-on simulation	Risk-free practice, skill enhancement, feedback integration	High cost, limited access in lower-income settings
AR simulation	Overlaying anatomical structures during live procedures	Spatial awareness, real-time guidance	Hardware dependency, cognitive overload risks
CADe	Real-time polyp detection during colonoscopy	Increased adenoma detection rates, real-time alerts	Technology dependency, requires training for optimal use
Generative AI and large language models	Educational support, training assistance, clinical decision support	Enhanced learning tools, accessible information processing	Data dependency, bias risks if datasets are non-diverse
AI-enhanced endoscopic imaging	Improved lesion detection and classification	Enhanced diagnostic accuracy, real-time feedback	Technology reliance, availability limited to certain centres
EUS with AI	Improved lesion identification in pancreatic and GI imaging	Enhanced diagnostic clarity, real-time feedback	Cost and technology barriers, requires specialised training

Quality of evidence 

The quality of evidence across the included literature is variable, reflecting a spectrum of methodological designs ranging from RCTs to narrative reviews, pilot studies, and observational reports. This heterogeneity means that there is certainly room for high-quality evidence, such as longitudinal multicentre studies. In the future, as we gather more high-quality data, our perceptions and attitudes will evolve about AI’s role in GI training. 

*High-Quality Evidence: RCTs and Meta-Analyses* 

Multiple RCTs (e.g., Huang et al., 2021; Lau et al., 2024; Vilmann et al., 2018; Cohen et al., 2006) provide some of the strongest evidence, due to their methodological rigour, use of control groups, and standardised outcomes such as adenoma detection rates, procedural time, and learning curve reduction. These trials demonstrate statistically significant improvements in trainee performance and patient outcomes when AI tools such as CADe systems are used. Their internal validity is strengthened by randomisation and prospective data collection, although external validity remains somewhat limited by relatively small sample sizes and specific institutional contexts. 

Likewise, meta-analyses and systematic reviews (Khan et al., 2019; Dhali et al., 2025; Gong et al., 2024) provide aggregated insights across multiple studies, enhancing the overall weight of evidence. For instance, Dhali et al. (2025) systematically reviewed AI-assisted capsule endoscopy, identifying consistent improvements in lesion detection. However, even these high-level studies may include primary literature with design or reporting limitations, potentially introducing bias into the pooled results. 

Moderate Evidence: Observational and Pilot Studies 

Studies such as those by Yuan et al. (2024) and Quan et al. (2022) add real-world applicability to the evidence base, evaluating AI tools like anatomical classification during EGD or real-time polyp detection in multi-centre settings. These studies report high diagnostic accuracy and clinical utility, contributing valuable external validity. However, their retrospective or non-randomised nature increases susceptibility to selection bias and confounding. Moreover, they often lack control groups, making it difficult to attribute improvements solely to the AI intervention. 

Low-Quality Evidence: Narrative Reviews and Perspective Articles 

Narrative reviews (Kang et al., 2025; Campion et al., 2024; Araújo et al., 2025; Ahmad et al., 2023) and expert perspectives (Ahmed et al., 2024; Ramoni et al., 2025) play an important role in identifying conceptual frameworks, theoretical benefits, and anticipated challenges related to AI implementation in education and diagnostics. These papers are useful for hypothesis generation and highlighting under-explored areas such as human-AI interaction and cognitive biases. However, their lack of systematic methodology, risk of author bias, and absence of empirical data place them at the lower end of the evidence hierarchy. 

Supplementary Evidence: Survey and Dataset Readiness Studies 

Survey-based research (Kader et al., [22]) offers insight into clinician perceptions and barriers to AI adoption, which is important for practical implementation and contributes towards specialists’ outlook of future directions of AI in gastroenterology, and is therefore important to include. However, this form of evidence is inherently subjective and lacks clinical outcome measures. Similarly, the focus on dataset readiness (Elamin et al., [25]) is critical for informing future AI model development and validation, but does not directly evaluate educational or clinical efficacy. 

**Figure 2:**
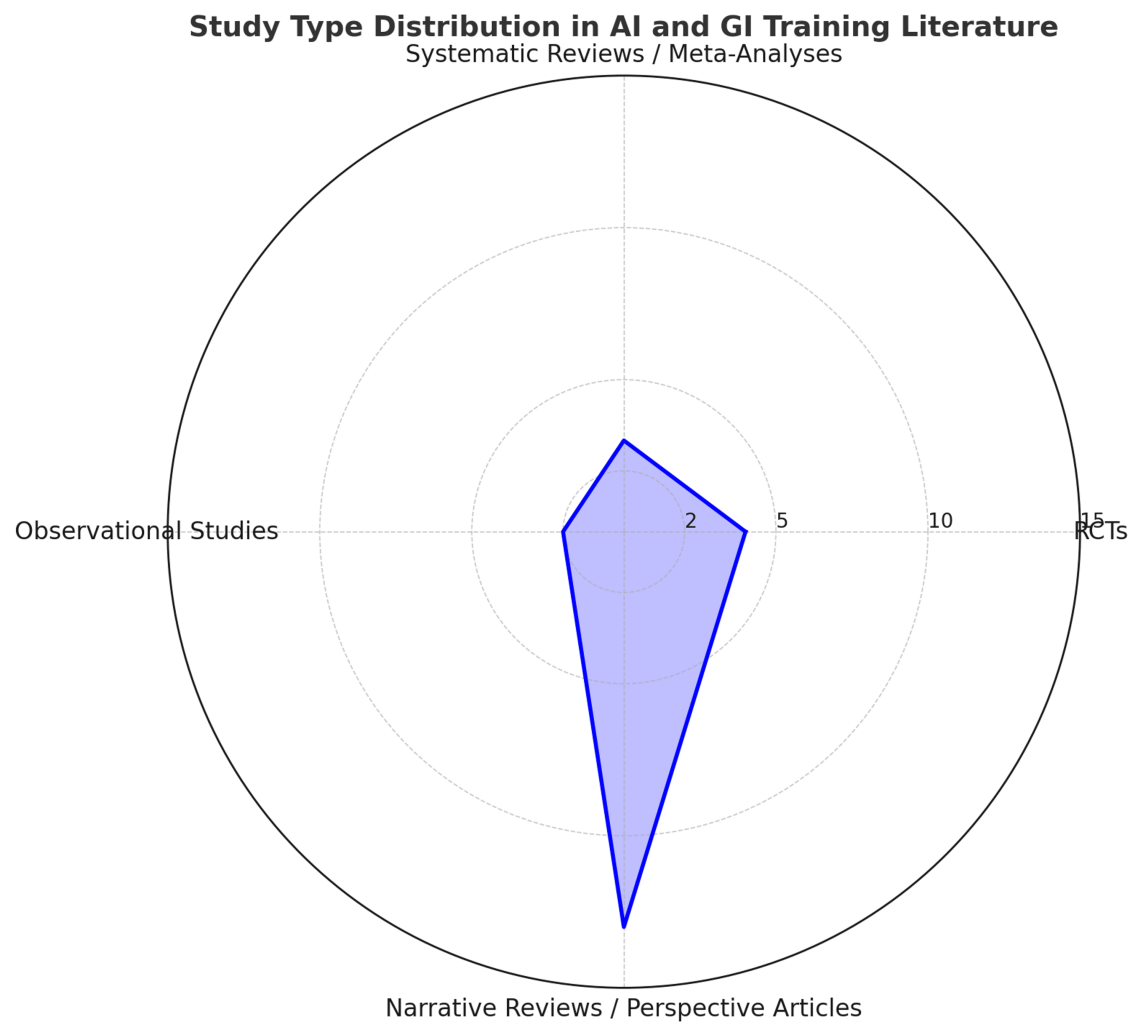
A radar chart on composition of study types showing comparative prominence of narrative reviews

In summary, the current evidence base is promising but uneven. Strong findings from RCTs and meta-analyses are offset by a predominance of narrative accounts and early-phase studies. Figure 2 shows a radar chart on the composition of study types showing the comparative prominence of narrative reviews. 

Limitations 

*Dataset Bias and Overfitting* AI models in gastroenterology are often trained on datasets that may not adequately represent diverse patient populations, increasing the risk of bias and reducing generalisability. This was noted in the context of paediatric endoscopy, where variation in anatomy and limited training data may compromise model accuracy (Dhaliwal and Walsh) [23]. The British Society of Gastroenterology AI Task Force also emphasises that many current models lack sufficient external validation, particularly across varying demographic and clinical contexts [22]. 

*Ethical and Cognitive Concerns* We have previously discussed at greater length concerns about over-reliance on AI may erode critical clinical judgement, particularly among trainees. As AI increasingly supports procedural and diagnostic tasks, clinicians risk becoming passive overseers rather than active decision-makers, especially in non-routine or ambiguous cases [7]. This sentiment is echoed by the Canadian Association of Gastroenterology, which highlights the importance of preserving cognitive engagement and critical thinking as AI tools become more prevalent in training [25]. 

Human-AI Interaction Challenges 

Campion et al. identify several psychological risks associated with AI-assisted endoscopy that may impact clinical performance and trainee development [6]. These include automation bias, where users may over-rely on AI outputs, potentially leading to diagnostic oversight; alarm fatigue, resulting from frequent or low-specificity alerts that desensitise users and increase the risk of missing critical warnings; and algorithm aversion, where witnessing an AI error leads to a loss of trust in the system, discouraging future use even when the tool is otherwise effective. These cognitive challenges highlight the importance of well-designed human-AI interfaces and comprehensive user training to ensure safe and effective integration of AI into clinical practice.

Lack of Longitudinal Evaluation 

While randomised trials such as those by Vilmann et al. and Lau et al. demonstrate short-term gains in trainee performance, few studies assess long-term retention of skills once AI support is removed [5,13]. The JCAG consensus (2024) also points to concerns about AI-assisted learning persisting over time or translating to clinical independence [25]. 

Need for Standardisation and Validation 

Kader et al. emphasise the absence of universally accepted standards for validating AI tools in gastroenterology [22]. Without clear protocols for assessing safety, effectiveness, and integration into clinical workflows, AI implementation remains fragmented and prone to variability in educational impact. 

Future directions 

The integration of AI in gastroenterology training is still in its developmental phase, but it shows promise for significant advancements. As technology continues to evolve, several emerging trends are anticipated to shape the future of AI-driven education in GI training. These include AR integration, predictive modelling, and adaptive learning, and the drive towards global standardisation of AI-based training protocols. 

Augmented Reality Integration 

AR is anticipated to play a transformative role in endoscopy training, offering immersive, real-time overlays of anatomical structures during live procedures. Unlike traditional VR systems, AR enables dynamic lesion localisation and contextual visualisation, thereby improving spatial awareness and procedural accuracy. Bhushan et al. (2018) demonstrated significant gains in spatial understanding and skill acquisition when trainees utilised AR platforms, suggesting that AR could serve as a vital bridge between simulation-based learning and real-world clinical application [12]. This makes AR an ideal complement to AI-powered diagnostic systems, reinforcing technical confidence in high-stakes environments. 

Predictive Modelling and Adaptive Learning 

AI-driven predictive modelling and adaptive learning systems represent a key frontier in personalised medical education. These tools can analyse performance data to identify learning gaps, forecast procedural errors, and adapt training content to the individual’s progress. For instance, AI-guided biopsies in IBD have demonstrated improved detection of remission without mucosal sampling, offering a model for how predictive analytics can guide decision-making in real time (Ahmad et al., 2023) [24]. Applied to education, such systems could evolve into intelligent tutoring platforms, providing real-time feedback, procedural adjustments, and scenario customisation based on the trainee’s actions-ensuring that skill acquisition is both targeted and responsive. 

Global Standardisation of AI-Based Training Protocols 

Despite promising advancements, a significant barrier to widespread AI adoption remains the lack of standardised training protocols. In a recent survey, 92% of participants cited the absence of clear guidelines as the primary obstacle to implementing AI in routine clinical practice [22]. This disparity in training exposure and platform accessibility leads to inconsistent learning outcomes across institutions. Studies such as Yuan et al. (2024) suggest that AI systems capable of identifying anatomical landmarks can help reduce operator-dependent variability and promote standardisation [9]. Additionally, evidence from Cohen et al. (2006) indicates that AI-enhanced virtual endoscopy labs can significantly improve clinical competencies across diverse training environments [3]. 

To ensure equitable access and uniformity in training, international regulatory bodies such as the World Endoscopy Organisation (WEO) and the American Society for Gastrointestinal Endoscopy (ASGE) should prioritise the development of global certification frameworks and consensus-based protocols. Survey respondents from the UK also strongly endorsed this direction, with 96% emphasising the need for identifying AI research priorities and 93% supporting the creation of clinical adoption guidelines [22]. 

Overcoming Challenges 

While enthusiasm for AI integration is high, concerns about accountability (85%) and algorithmic bias (82%) remain substantial hurdles [22]. Furthermore, barriers to research-particularly limited funding (82%) and insufficient annotated data (76%), must be addressed to sustain innovation. The British Society of Gastroenterology (BSG) AI Task Force, and similar organisations, are therefore encouraged to prioritise support for multi-centre trials (91%) and establish robust infrastructures for data sharing and algorithm validation [22]. 

The integration of AI into gastroenterology is poised to revolutionise both clinical practice and medical education. Survey results indicate that quality improvement in endoscopy (97%) and enhanced diagnostic capabilities (92%) are perceived as the most beneficial clinical applications of AI, while the top research priority identified is real-time endoscopic image diagnosis (95%) [22]. These insights underscore the growing momentum towards harnessing AI to elevate diagnostic precision and streamline training in endoscopic procedures.

Recommendations for future research 

Multi-Centre Longitudinal Studies 

Future research should ideally consist of large-scale, multi-centre RCTs with extended follow-up periods to assess the long-term retention of AI-enhanced skills. Current studies predominantly focus on short-term outcomes, leaving critical questions about skill durability and clinical independence relatively uncertain. Longitudinal cohort studies tracking trainee performance over 2-5 years post-training would provide essential insights into the sustained benefits of AI integration. 

Standardised Assessment Frameworks 

Development of validated competency assessment tools specific to AI-enhanced training is urgently needed. Research should establish standardised metrics for evaluating diagnostic accuracy, procedural confidence, and clinical decision-making in AI-assisted environments. This includes creating objective performance indicators that can be universally applied across institutions and training programs. 

Personalised Learning Algorithms 

Investigation into adaptive AI systems that customise training content based on individual learning patterns, skill gaps, and progression rates represents a significant research opportunity. Machine learning algorithms could analyse trainee performance data to optimise educational pathways, potentially reducing training time while improving outcomes. 

Comparative Effectiveness Research 

Head-to-head comparisons between different AI training modalities (VR vs. AR vs. hybrid approaches) are essential to guide resource allocation and curriculum design. Research should also compare AI-enhanced training against traditional methods using standardised patient outcomes and cost-effectiveness analyses. 

Equity and Accessibility Studies 

Research addressing disparities in AI access across training programs, particularly in resource-limited settings, is critical. Studies should explore cost-effective implementation strategies, mobile-based solutions, and partnerships that could democratise access to AI-enhanced training globally. 

Human-AI Interaction Optimisation 

Investigation into cognitive ergonomics of AI-assisted training, including studies on alarm fatigue, automation bias, and optimal feedback mechanisms. Research should focus on designing AI interfaces that enhance rather than replace critical thinking skills. 

Ethical Framework Development 

Systematic research into the ethical implications of AI dependency in medical training, including studies on maintaining clinical judgment, accountability frameworks, and patient safety considerations in AI-integrated healthcare environments. 

Recommended Study Designs 

Future research should employ a range of robust methodologies to comprehensively evaluate the impact of AI in gastroenterology training. Cluster randomised trials comparing AI-enhanced programmes across multiple institutions would provide high-quality evidence on effectiveness and scalability. Mixed-methods studies that combine quantitative outcomes with qualitative insights from trainees and educators could offer a more nuanced understanding of user experience and educational value. Implementation science research is also essential to explore the real-world barriers and facilitators to AI adoption within diverse clinical settings. Additionally, economic evaluations assessing cost-effectiveness and return on investment would help justify resource allocation and guide policy decisions. Finally, registry studies tracking long-term career outcomes of AI-trained versus traditionally trained gastroenterologists would offer valuable insight into the lasting impact of AI integration on professional development and clinical performance.

## Conclusions

This literature review highlights the transformative role of AI in GI training, revealing substantial improvements in simulation-based learning, diagnostic decision support, and procedural skill acquisition. Across 22 studies published between 2005 and 2025, AI consistently enhanced learning outcomes, increased diagnostic accuracy, and accelerated procedural skills among trainees, demonstrating how AI can enhance the educational experience for GI trainees across diverse training environments.

To ensure responsible and inclusive implementation, coordinated efforts are needed-ranging from global standardisation and multi-centre RCTs to the use of diverse datasets that reflect a broad patient population. Future training programmes should adopt AI as an adaptive educational partner, offering personalised, responsive feedback that enhances, rather than replaces, core competencies. This inclusive and hybrid model of education will cultivate an environment that merges technology with human insight, ensuring that scientific advancements such as AI and simulation are truly used to improve trainees' skills and patient safety simultaneously.
